# Sex differences in drug-induced osteoporosis: a pharmacovigilance study based on the FAERS database

**DOI:** 10.3389/fpubh.2025.1630412

**Published:** 2025-07-24

**Authors:** Lu Liu, Yongjia Song, Xiaoyu Liu, Bangguo Song, Min Song

**Affiliations:** Clinical College of Traditional Chinese Medicine, Gansu University of Chinese Medicine, Lanzhou, China

**Keywords:** drug-induced osteoporosis, FAERS, sex-specific risk, mGPS, BCPNN, time-to-onset analysis, pharmacovigilance

## Abstract

**Background:**

Osteoporosis is a prevalent condition globally, often linked to a significant risk of fractures. Drug-induced osteoporosis (DIOP) is an increasingly recognized adverse effect of various medications, but the sex-specific risks and time-to-onset patterns remain inadequately understood. Addressing these gaps in knowledge is critical to improving patient safety and pharmacovigilance.

**Objective:**

This study aimed to explore sex-related differences in DIOP, identify high-risk medications, and assess the onset patterns of osteoporosis-related adverse events by analyzing data from the FDA Adverse Event Reporting System (FAERS) and validating the findings using the Canada Vigilance Adverse Reaction Online Database (Canada Vigilance ADR).

**Methods:**

We analyzed adverse event reports from the FAERS database covering the period from Q1 2004 to Q4 2024. Drugs were standardized using the RxNorm drug terminology system, and adverse events were matched to MedDRA 27.1. Disproportionality analysis was conducted using Reporting Odds Ratio (ROR), Multi-item Gamma Poisson Shrinker (MGPS), and Bayesian Confidence Propagation Neural Network (BCPNN) methods. To validate our findings, we performed external validation using the Canada Vigilance ADR database. Stratified analyses by sex were performed to assess differences in drug-osteoporosis associations.

**Results:**

A total of 236,928 osteoporosis-related reports were identified, with 64.6% of the reports coming from females. We identified 68 drugs associated with DIOP, including 15 male-specific and 26 female-specific potential risk drugs. Notable drugs such as tenofovir disoproxil and esomeprazole were linked to both sexes. Drugs like upadacitinib exhibited early-onset failure patterns, while others like tenofovir demonstrated cumulative risk patterns over prolonged use. External validation with the Canada Vigilance ADR confirmed 32 drugs with potential osteoporosis risks.

**Conclusions:**

This study highlights important sex-specific differences in the risk of drug-induced osteoporosis and underscores the need for targeted pharmacovigilance strategies. The findings contribute to a more personalized approach to drug safety, promoting more informed decision-making regarding medication use in osteoporosis-prone populations.

## 1 Introduction

Osteoporosis (OP) is a common skeletal disorder worldwide, characterized by reduced bone mineral density and increased bone fragility, which significantly elevates the risk of fractures (1). According to data from the Global Burden of Disease (GBD), osteoporosis has become a leading cause of fractures among older adult populations, with this trend continuing to rise globally, particularly in high-income countries (2). Although the progression of osteoporosis can be effectively mitigated through pharmacological treatments, dietary adjustments, and physical exercise, drug-related adverse effects—especially drug-induced osteoporosis (DIOP)—remain a critical concern in clinical practice (3).

Drug-induced osteoporosis refers to bone loss and skeletal fragility resulting from long-term or inappropriate use of certain medications (4). Numerous drugs, notably glucocorticoids, antiepileptic agents, and hormone therapies, have been closely associated with the onset of DIOP (5). Among them, glucocorticoids are the most widely recognized risk factor; however, the impact of other drugs on bone health, particularly their differential effects across sexes, is not yet fully understood.

In recent years, DIOP has attracted increasing attention in clinical and public health fields. Some studies have indicated that males and females may differ significantly in the incidence, pathophysiological mechanisms, and drug responses related to DIOP. These sex-specific differences in bone metabolism, hormonal levels, and drug pharmacokinetics may influence how medications affect bone health in men and women (6). Nevertheless, most existing studies have focused on the general impact of drugs on bone density, with limited exploration of sex differences in drug-induced bone loss (7).

To address this gap, the present study utilizes pharmacovigilance data from the U.S. Food and Drug Administration's Adverse Event Reporting System (FAERS) to investigate sex-based differences in DIOP (8). Disproportionality analysis (DPA) and other signal detection methods are employed to identify potential drug-related osteoporosis risk signals. Through this study, we aim to provide a more nuanced understanding of sex-specific risks in drug safety assessments, thereby assisting clinicians in identifying and managing DIOP more effectively and offering evidence-based guidance for personalized medication strategies.

## 2 Materials and methods

### 2.1 Data sources and processing

The adverse event data for this study were obtained from the FDA Adverse Event Reporting System (FAERS) database (https://fis.fda.gov/extensions/FPD-QDE-FAERS/FPD-QDE-FAERS.html). This publicly accessible database has been available since 2004. We retrieved adverse event reports from the ASCII-format FAERS database covering the period from the first quarter of 2004 to the fourth quarter of 2024.

For entries with identical case IDs (report identifiers), only the most recent report, based on the submission date, was retained, and duplicate records were removed. Drug names were standardized using RxNorm version 2024-04-01 to ensure consistency across the FAERS dataset. The Preferred Terms (PTs) for osteoporosis/osteopenia were identified using version 27.1 of the Medical Dictionary for Regulatory Activities (MedDRA 27.1), ensuring that the classification of adverse events was aligned with current regulatory standards.

After standardizing the drug and adverse event terms, we extracted reports of suspected (primary suspect, PS) drugs associated with osteoporosis/osteopenia. These reports were then characterized by sex, age, body weight, indication, country of report, and clinical outcome. To ensure the accuracy of the study, external validation was conducted with the Canada Vigilance Adverse Reaction Online Database (Canada Vigilance ADR).

### 2.2 Signal detection algorithm

In this study, disproportionality analysis (DPA), a widely used pharmacovigilance data mining method, was employed to detect potential signals of osteoporosis/osteopenia-related adverse events (AEs). Disproportionality analysis evaluates the association between drugs and AEs by comparing observed reporting frequencies in exposed vs. unexposed populations using a 2 × 2 contingency table (see Supplementary Material 1). To enhance the stability and reliability of signal detection, we adopted a composite strategy incorporating three established DPA algorithms to identify drug-induced osteoporosis signals from the FAERS database. The methods are described as follows: Reporting Odds Ratio (ROR): ROR is the most commonly used DPA method and calculates the strength of association between a drug and an AE using 2 × 2 contingency tables. A signal is considered positive if the lower limit of the 95% confidence interval (Lower CI) of the ROR exceeds 1, and the number of reports is ≥3 (9). Multi-item Gamma Poisson Shrinker (MGPS): MGPS is a Bayesian shrinkage algorithm recommended by the U.S. FDA. It evaluates drug-event associations using the Empirical Bayes Geometric Mean (EBGM). A signal is defined as positive when EB05 (the lower bound of the 95% confidence interval for EBGM) exceeds 2 (10). Bayesian Confidence Propagation Neural Network (BCPNN): The core metric in BCPNN is the Information Component (IC), which quantifies the association strength. A signal is considered positive if IC025 (the lower bound of the 95% confidence interval for IC) is >0 (11).

To ensure robustness and clinical credibility in detecting sex-specific potential signal drugs, only those meeting the positive criteria for all three algorithms simultaneously (i.e., ROR-positive + MGPS-positive + BCPNN-positive) were retained as high-confidence signals.

Furthermore, effective signals were considered novel adverse event signals if they were not listed in the FDA-approved drug labeling (https://www.accessdata.fda.gov/scripts/cder/daf/index.cfm).

**Figure 1:**
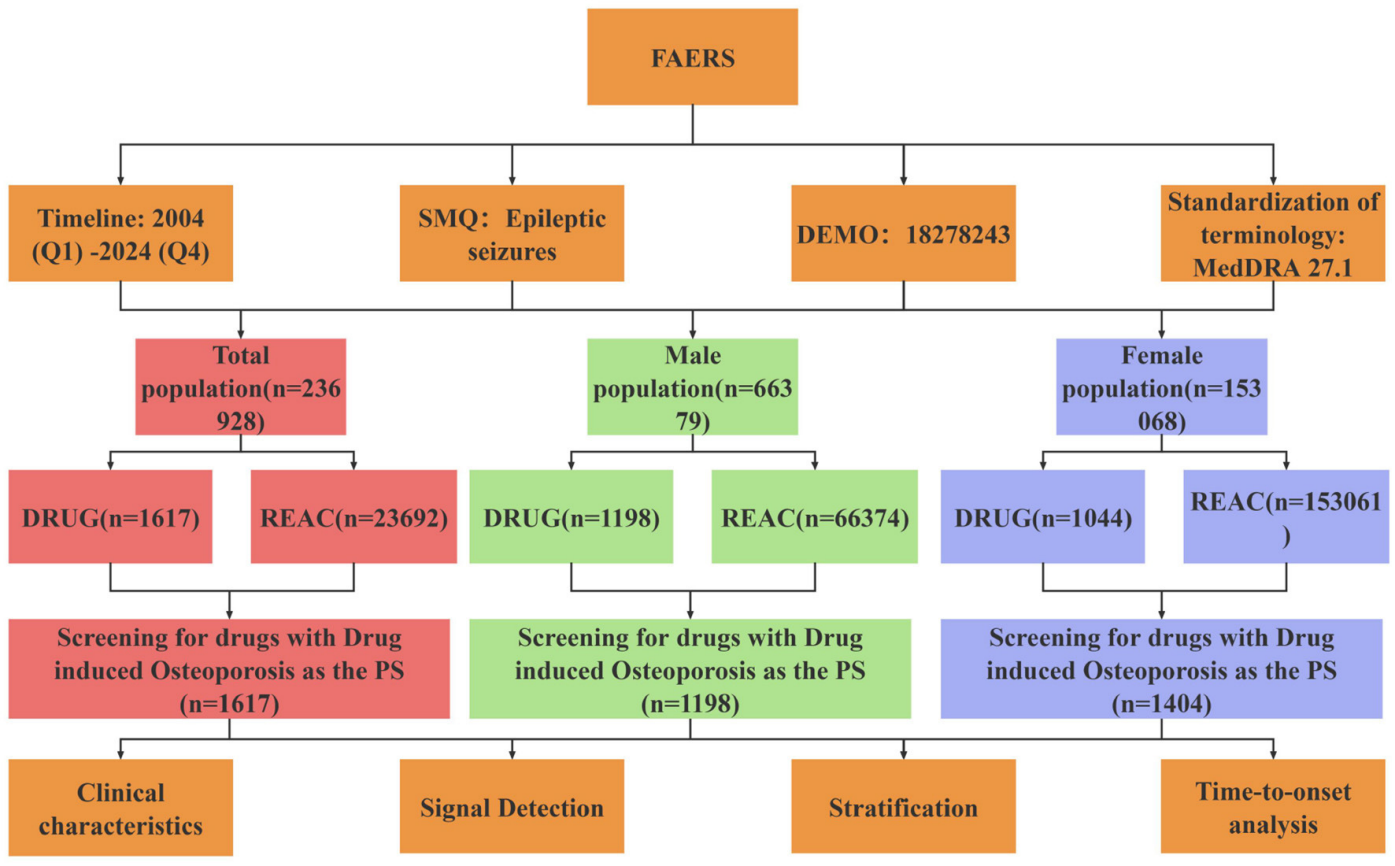
Flow chart of drug-related osteoporosis signal mining. SMQ, standardized MedDRA query; DEMO, demographic information; DRUG, drug information; REAC, reaction information.

All data processing and statistical analyses were conducted using R version 4.4.0 and Microsoft Excel. The data extraction and signal detection workflow is illustrated in Figure 1.

## 3 Results

### 3.1 Basic characteristics of osteoporosis-related adverse events

**Figure 2:**
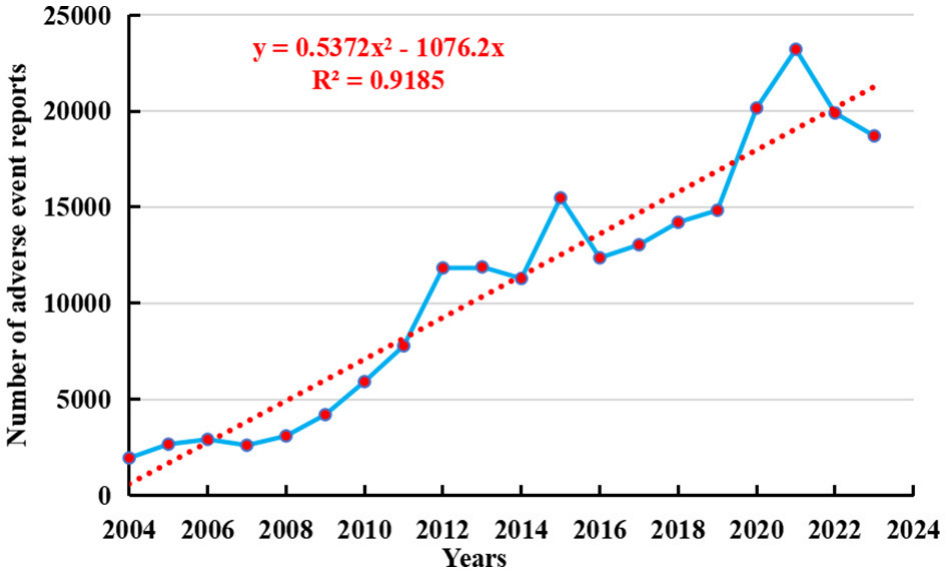
Number of annual reports of drug-related osteoporosis.

As of the fourth quarter of 2024, a total of 236,928 adverse event reports related to osteoporosis had been recorded in the FAERS database. From the first quarter of 2004 to the fourth quarter of 2024, the number of adverse events reported with osteoporosis as the preferred term has shown a yearly increase, peaking in 2021 with 23,213 cases (see Figure 2). To further forecast future reporting trends, a polynomial regression model was fitted to the data. The resulting curve exhibited a rapidly increasing trend, with a coefficient of determination (*R*^2^) of 0.9185, indicating that the model explains 91.85% of the variability in the data and thus provides valuable predictive insight.

Table 1 summarizes the characteristics of patients involved in osteoporosis-related adverse events. Among all reports, 64.6% were from female patients, 28.0% from males, and 7.4% had unspecified sex. Regarding age distribution, individuals aged 18–65 years accounted for the largest proportion (29.06%) of reports with known age, followed by those aged 65–85 years (27.54%). With respect to body weight, most adverse events were reported in individuals weighing 50–69 kg (10.34%), although 74.48% of the reports lacked weight information.

**Table 1:** Baseline characteristics of people with drug-related osteoporosis.

**Characteristics**	**Case numbers**	**Case proportion (%)**
Number of events	*N* = 236,928	–
**Gender**		
Male	66,379	28.0
Female	153,068	64.6
Miss	17,481	7.4
**Age**		
Median age	65	
< 18	2,755	1.16
18–65	68,850	29.06
65–85	65,249	27.54
>85	9,058	3.82
Miss	91,016	38.42
**Weight (kg)**		
< 50	6,632	2.80
50–69	24,504	10.34
70–89	18,701	7.89
≥90	10,634	4.49
Miss	176,457	74.48
**Top five indication**		
Product used for unknown indication	30,195	12.74
HIV infection	23,467	9.90
Rheumatoid arthritis	20,004	8.44
Plasma cell myeloma	7,805	3.29
Multiple sclerosis	7,564	3.19
**Top five reported countries**		
United States	146,091	61.66
Japan	17,629	7.44
Canada	12,462	5.26
France	7,217	3.05
Germany	6,825	2.88

Interestingly, the most frequently reported indication was “Product used for unknown indication” (12.74%), followed by “HIV infection” (9.90%) and “Rheumatoid arthritis” (8.44%). In terms of geographic distribution, the majority of DIOP-related adverse events were reported in high-income countries, with the United States accounting for 61.66% of cases, followed by Japan (7.44%) and Canada (5.26%).

### 3.2 Analysis of drugs with disproportional reporting signals in the overall population

**Figure 3:**
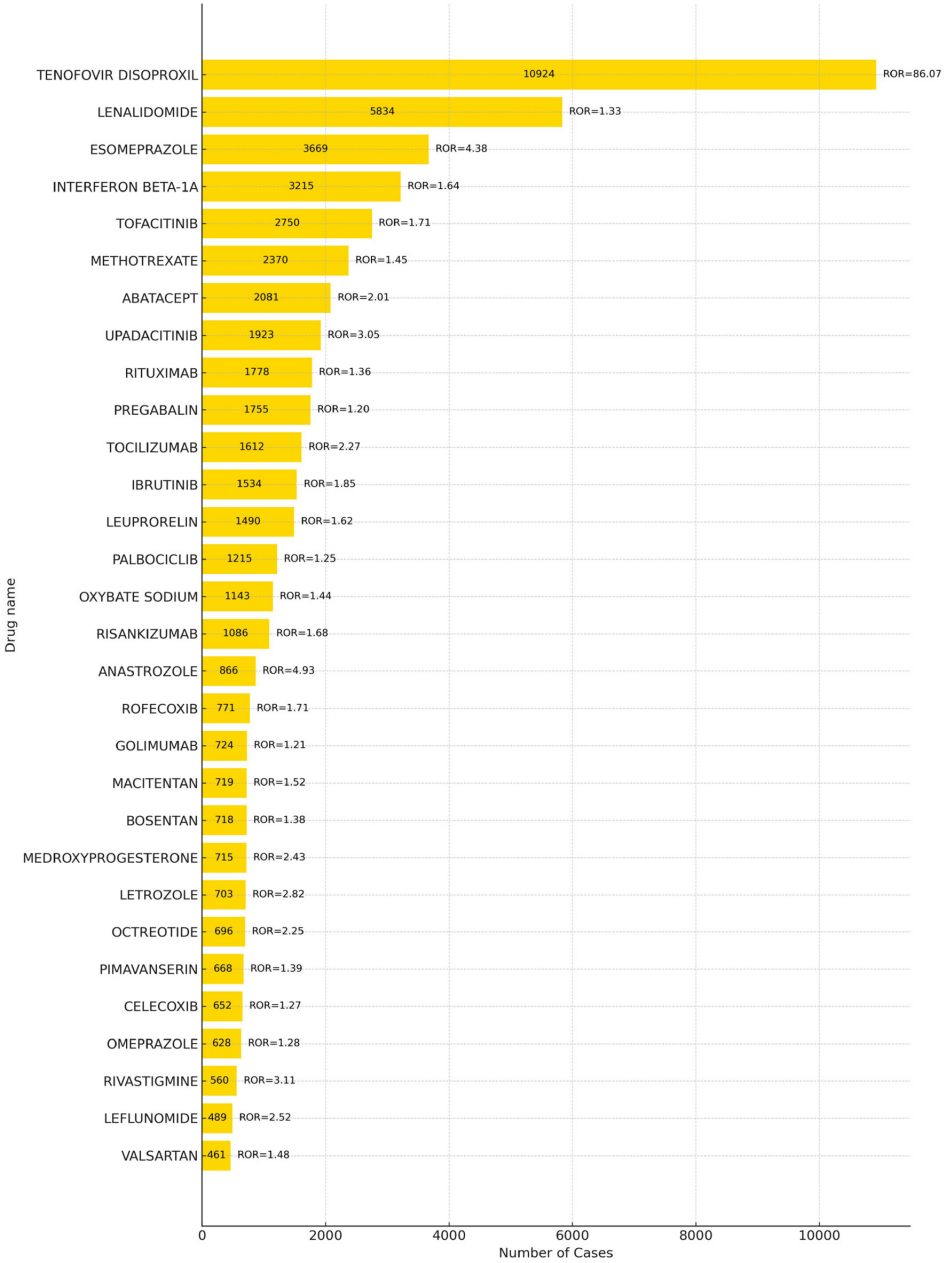
Top 30 dangerous drugs for drug-induced osteoporosis.

A total of 68 drugs were associated with adverse events related to drug-induced osteoporosis (DIOP), with the top 30 drugs presented in Figure 3. The five most frequently reported drugs were tenofovir disoproxil (10,924 reports), lenalidomide (5,834), esomeprazole (3,669), interferon beta-1A (3,215), and tofacitinib (2,750).

Further comparison with the official drug labels revealed that 50 of these drugs—such as tenofovir disoproxil^*^, lenalidomide^*^, interferon beta-1A^*^, abatacept^*^, and upadacitinib^*^–did not explicitly mention osteoporosis-related adverse reactions in their prescribing information. ^*^Indicates a new signal of adverse reaction to osteoporosis not mentioned in the WHO drug instructions. These findings represent newly identified safety signals and warrant further clinical attention. Detailed information is provided in Supplementary Material 2.

### 3.3 Analysis of sex-specific drugs with disproportional reporting signals

We further applied Bayesian-adjusted disproportionality analysis (B-correction), and identified 20 potential signal drugs in the male population, as shown in Table 2. The most frequently reported drugs included tenofovir disoproxil (*N* = 7,381), esomeprazole (*N* = 688), upadacitinib (*N* = 368), adefovir (*N* = 259), and tafamidis (*N* = 200). In the female population, a total of 28 risk drugs were identified, among which tenofovir disoproxil (*N* = 3,188), esomeprazole (*N* = 2,299), upadacitinib (*N* = 1,507), tocilizumab (*N* = 1,380), and ibrutinib (*N* = 880) were the most frequently reported.

**Table 2:** Drug related osteoporosis reported in FAERS.

**Gender**	**Drug**	**Number of adverse events**	**ROR.95.Cl**.	**EBGM.EBGM05**.	**IC.IC025**.
Male (*N* = 20)	Tenofovir disoproxil	7,381	150.86 (145.5–156.42)	56.83 (55.14)	5.83 (5.79)
	Esomeprazole	688	3.4 (3.15–3.67)	3.3 (3.09)	1.72 (1.61)
	Upadacitinib^*^	368	2.77 (2.49–3.07)	2.71 (2.48)	1.44 (1.28)
	Adefovir	259	27.92 (24.31–32.06)	21.83 (19.44)	4.45 (4.25)
	Tafamidis^*^	200	2.52 (2.19–2.9)	2.48 (2.2)	1.31 (1.1)
	Radium RA 223 dichloride^*^	163	4.13 (3.53–4.83)	4 (3.5)	2 (1.77)
	Rivastigmine^*^	136	2.36 (1.99–2.8)	2.33 (2.02)	1.22 (0.97)
	Lamivudine^*^	118	2.7 (2.25–3.24)	2.65 (2.27)	1.41 (1.14)
	Deflazacort	80	5.2 (4.15–6.51)	4.98 (4.13)	2.32 (1.99)
	Imiglucerase^*^	77	5.23 (4.16–6.58)	5.01 (4.13)	2.32 (1.99)
	Triptorelin	66	3.54 (2.77–4.52)	3.45 (2.81)	1.79 (1.43)
	Asfotase alfa^*^	57	3.07 (2.35–3.99)	3 (2.41)	1.59 (1.2)
	Aclidinium^*^	52	3.64 (2.76–4.8)	3.54 (2.81)	1.82 (1.42)
	Velaglucerase alfa^*^	48	7.36 (5.49–9.87)	6.91 (5.4)	2.79 (2.36)
	Rasagiline^*^	45	3.62 (2.68–4.87)	3.52 (2.74)	1.82 (1.38)
	Etelcalcetide^*^	31	3.09 (2.16–4.42)	3.03 (2.24)	1.6 (1.08)
	Amifampridine^*^	25	3.76 (2.52–5.6)	3.65 (2.61)	1.87 (1.29)
	Ciclesonide	23	4.24 (2.79–6.43)	4.1 (2.89)	2.04 (1.43)
	Tenofovir	21	5.8 (3.73–9)	5.52 (3.82)	2.47 (1.83)
	Etidronic acid^*^	8	33.69 (15.07–75.32)	25.25 (12.88)	4.66 (3.57)
Female (*N* = 28)	Tenofovir disoproxil	3,188	71.79 (68.25–75.52)	33.92 (32.52)	5.08 (5.02)
	Esomeprazole^*^	2,299	3.99 (3.82–4.16)	3.77 (3.64)	1.92 (1.85)
	Upadacitinib^*^	1,507	2.9 (2.76–3.06)	2.8 (2.68)	1.49 (1.41)
	Tocilizumab^*^	1,380	2.33 (2.21–2.46)	2.27 (2.17)	1.19 (1.11)
	Ibrutinib	880	2.41 (2.26–2.58)	2.36 (2.23)	1.24 (1.14)
	Anastrozole	839	4.27 (3.98–4.58)	4.05 (3.82)	2.02 (1.91)
	Letrozole	669	2.42 (2.24–2.61)	2.36 (2.21)	1.24 (1.12)
	Prednisone	532	2.2 (2.02–2.4)	2.16 (2.01)	1.11 (0.98)
	Octreotide^*^	481	2.7 (2.47–2.96)	2.63 (2.43)	1.39 (1.26)
	Leflunomide^*^	407	2.46 (2.23–2.72)	2.41 (2.21)	1.27 (1.12)
	Rivastigmine	396	3.3 (2.98–3.65)	3.18 (2.92)	1.67 (1.52)
	Asfotase alfa^*^	191	2.73 (2.37–3.16)	2.66 (2.36)	1.41 (1.2)
	Pioglitazone	161	3.5 (2.98–4.1)	3.37 (2.95)	1.75 (1.52)
	Aclidinium^*^	156	3.95 (3.36–4.65)	3.78 (3.3)	1.92 (1.68)
	Imiglucerase^*^	118	3.71 (3.08–4.46)	3.56 (3.04)	1.83 (1.56)
	Adefovir	96	17.31 (13.81–21.69)	13.81 (11.44)	3.79 (3.47)
	Rasagiline^*^	64	3.4 (2.64–4.37)	3.28 (2.65)	1.71 (1.35)
	Velaglucerase alfa^*^	52	4.06 (3.07–5.38)	3.88 (3.07)	1.96 (1.55)
	Indacaterol^*^	45	2.83 (2.1–3.82)	2.76 (2.15)	1.46 (1.03)
	Ciclesonide	42	2.87 (2.11–3.91)	2.79 (2.15)	1.48 (1.03)
	Entacapone^*^	32	3.04 (2.13–4.34)	2.95 (2.19)	1.56 (1.05)
	Carbidopa^*^	28	3.26 (2.23–4.77)	3.15 (2.29)	1.66 (1.11)
	Tenofovir	19	4.83 (3.03–7.71)	4.56 (3.09)	2.19 (1.52)
	Taliglucerase alfa^*^	14	3.82 (2.23–6.56)	3.66 (2.33)	1.87 (1.11)
	Gadoversetamide^*^	11	4.37 (2.37–8.06)	4.16 (2.49)	2.06 (1.19)
	Felbamate^*^	8	4.54 (2.22–9.31)	4.31 (2.36)	2.11 (1.11)
	Ferric hydroxide^*^	8	11.31 (5.33–23.99)	9.75 (5.2)	3.29 (2.25)
	Somatrem^*^	4	7.27 (2.58–20.46)	6.63 (2.79)	2.73 (1.36)

ROR, reporting odds ratio; CI, confidence interval.

* New signals of osteoporosis adverse reactions are not mentioned in the instructions.

By comparing with the corresponding drug labeling information, we found 13 novel signal drugs in males, including upadacitinib^*^, tafamidis^*^, radium Ra 223 dichloride^*^, rivastigmine^*^, and lamivudine^*^, with more than 100 reports each for these five drugs. In females, 18 novel signal drugs were detected, of which eight drugs—esomeprazole^*^, upadacitinib^*^, tocilizumab^*^, octreotide^*^, leflunomide^*^, asfotase alfa^*^, aclidinium^*^, and imiglucerase^*^–had more than 100 reports.

Importantly, none of these newly identified signal drugs explicitly mention osteoporosis-related risks in their package inserts. These findings warrant further clinical attention and post-marketing safety evaluation.

### 3.4 External validation with the Canadian database

**Figure 4:**
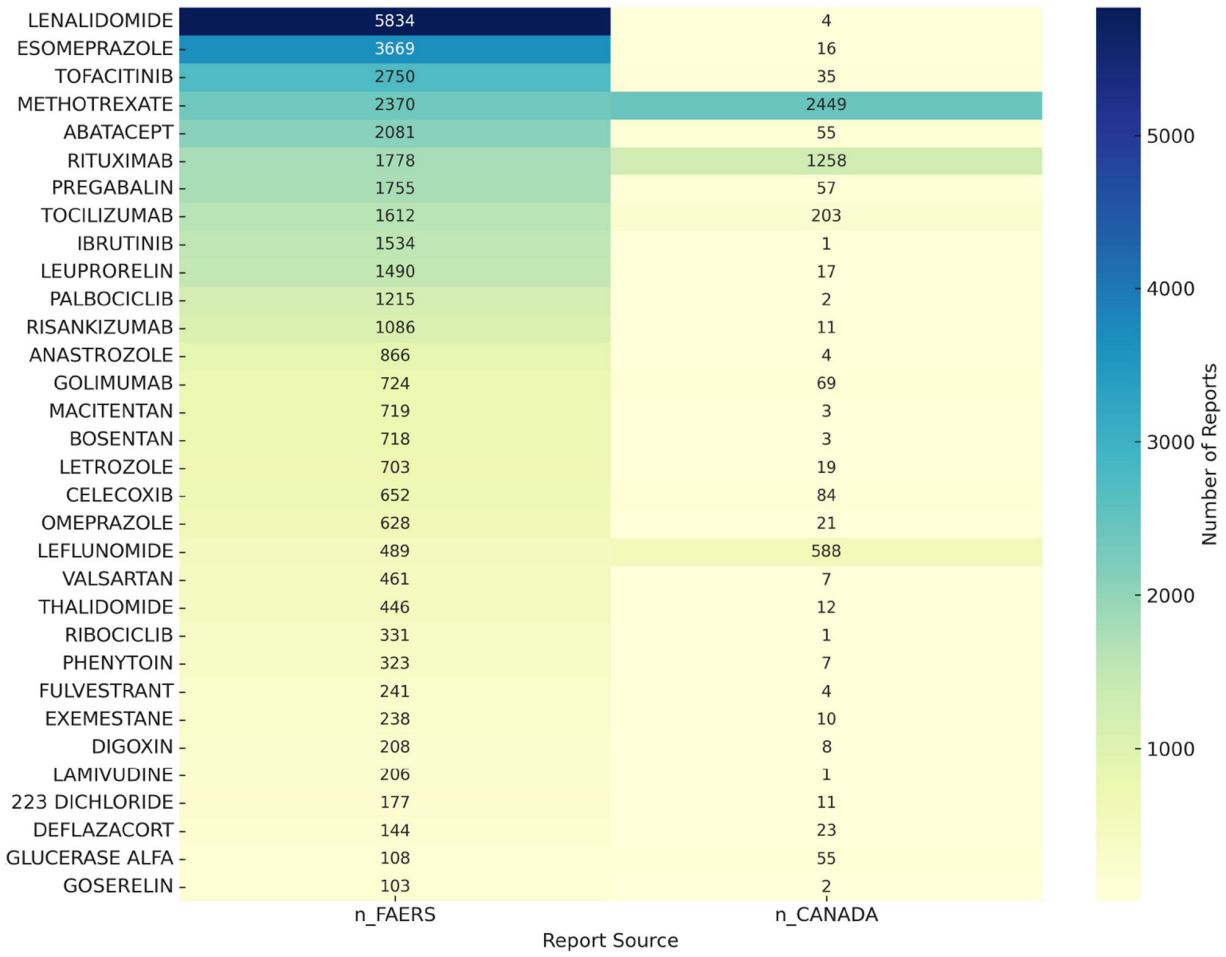
Heat map of osteoporosis risk drugs from the analysis of FAERS and CVAROD.

To further ensure the accuracy of our research findings, we conducted an additional analysis using the Canadian database for osteoporosis-related adverse events. We identified 2,021 drugs reported for osteoporosis-related adverse events in the Canadian database. A comparative analysis between the Canadian and FAERS databases revealed 32 drugs, including lenalidomide, esomeprazole, tofacitinib, methotrexate, and abatacept, that are associated with potential risks of osteoporosis. A heatmap illustrating these findings is shown in Figure 4.

### 3.5 Time-to-onset analysis

**Figure 5:**
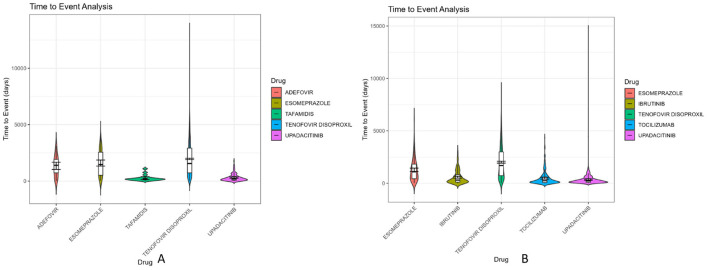
Violin diagram of drug induction time of the first five danger signals of men and women. **(A)** Male danger signal drugs; **(B)** Female danger signal drugs.

Time-to-onset (TTO) analysis plays a critical role in drug safety evaluation by identifying temporal risk windows and informing personalized medication strategies. In this study, we extracted TTO data from the FAERS database and focused on the top five potential signal drugs with the most reports in males and females, respectively. Violin plots were generated to visualize the distribution patterns (Figure 5), and Weibull distribution modeling was used to classify the time-dependent characteristics of adverse drug events (ADEs).

The Weibull model characterizes the shape of time-to-onset distribution using a shape parameter (β), which determines the failure pattern: Early failure (β < 1): Higher risk shortly after drug initiation, followed by a decline in event rate over time. Wear-out failure (β > 1): Increasing risk with prolonged drug exposure, suggestive of cumulative toxicity. Random failure (β ≈ 1): Constant risk throughout the treatment period.

Among males, the top five reported drugs were tenofovir disoproxil, esomeprazole, upadacitinib, adefovir, and tafamidis. Tenofovir disoproxil demonstrated the longest median TTO (1,555 days) and a shape parameter β = 1.18 (95% CI: 1.15–1.22), indicating a wear-out failure profile. A similar wear-out pattern was observed for esomeprazole (β = 1.32, 95% CI: 1.06–1.57). In contrast, upadacitinib showed an early failure profile (β = 0.99, 95% CI: 0.86–1.11), with the majority of ADEs occurring during the early treatment phase. Adefovir (β = 1.22, CI includes 1) and tafamidis (β = 1.10, CI includes 1) were classified as random failure patterns, indicating ADEs may occur unpredictably over time.

In the female group, the top five drugs included tenofovir disoproxil, esomeprazole, upadacitinib, tocilizumab, and ibrutinib. Tenofovir disoproxil again exhibited a long median TTO (1,698 days) and a consistent wear-out failure pattern (β = 1.21, 95% CI: 1.15–1.27). The other four drugs—upadacitinib (β = 0.87), tocilizumab (β = 0.76), and ibrutinib (β = 0.85)—had β values significantly below 1 with tight confidence intervals, confirming early failure profiles. The detailed results of the Weibull distribution modeling, including median TTO, scale parameters (α), and shape parameters (β) with 95% confidence intervals, are summarized in Table 3.

**Table 3:** Time to onset of drug related osteoporosis and Weibull distribution analysis.

**Gender**	**Drug**	**TTO (days)**		**Weibull distribution**		
		**Case reports**	**Median (day)**	**Scale parameter: α (95% CI)**	**Shape parameter: β (95% CI)**	**Type**
Male	Tenofovir disoproxil	7,381	1,555	2,077.46 (2,010.91–2,144.01)	1.18 (1.15–1.22)	Wear type fault curve
	Esomeprazole	688	1,464	1,721.20 (1,398.01–2,044.38)	1.32 (1.06–1.57)	Wear type fault curve
	Upadacitinib	368	194	303.79 (250.06–357.51)	0.99 (0.86–1.11)	Early failure
	Adefovir	259	1,402	1,419.22 (1,006.68–1,831.76)	1.22 (0.86–1.58)	Random failure curve
	Tafamidis	200	186	271.51 (154.05–388.97)	1.10 (0.73–1.47)	Random failure curve
Female	Tenofovir disoproxil	3,188	1,698	2,138.14 (2,031.55–2,244.73)	1.21 (1.15–1.27)	Wear type fault curve
	Esomeprazole	2,299	1,095	1,346.65 (1,159.96–1,533.35)	1.15 (1.01–1.29)	Wear type fault curve
	Upadacitinib	1,507	219	327.87 (294.46–361.27)	0.87 (0.82–0.93)	Early failure
	Tocilizumab	1,380	240	406.95 (336.93–476.98)	0.76 (0.69–0.83)	Early failure
	Ibrutinib	880	306	530.38 (437.36–623.49)	0.85 (0.75–0.94)	Early failure

TTO, time to onset; CI, confidence interval.

In summary, tenofovir disoproxil showed a consistent wear-out failure profile in both sexes, highlighting a cumulative risk over time and the need for sustained long-term monitoring. In contrast, drugs like upadacitinib, tocilizumab, and ibrutinib displayed early failure characteristics, underscoring the importance of close clinical surveillance during the early phases of treatment to mitigate the risk of acute onset adverse reactions.

## 4 Discussion

Drug-induced osteoporosis (DIOP) has emerged as a significant global public health issue, especially with the growing use of a wide range of medications (12). Although the association between drugs and osteoporosis has garnered increasing attention, sex-specific differences in the onset and progression of DIOP remain underexplored (13). This pharmacovigilance-based study, using post-marketing safety data from the FDA Adverse Event Reporting System (FAERS), systematically analyzed sex-related differences in DIOP and identified a series of potential risk signals associated with various drugs.

### 4.1 Susceptible populations and clinical management

This study further investigated the demographic characteristics of patients reporting DIOP-related adverse events. Among reports with known age, individuals aged 18–65 years accounted for the highest proportion (29.06%), followed by those aged 65–85 years (27.54%). In terms of body weight, patients in the 50–69 kg range made up 10.34% of reports, although 74.48% of cases lacked weight information. Body weight is a key factor influencing drug metabolism; however, due to the substantial proportion of missing data, it is difficult to comprehensively evaluate its role in the risk of DIOP (14). Therefore, future studies should aim to improve the completeness of data collection for critical variables such as weight to enhance the accuracy of risk assessments.

Regarding indications, DIOP-related adverse events were most commonly reported in patients with “unknown indication” (12.74%), followed by HIV infection (9.90%) and rheumatoid arthritis (8.44%). These conditions may contribute to bone density reduction through long-term medication use. For instance, patients with rheumatoid arthritis often undergo prolonged glucocorticoid therapy, which increases the risk of fractures (15). Similarly, individuals with HIV infection frequently use antiretroviral drugs that may disrupt bone metabolism, thereby elevating the risk of osteoporosis (16). In clinical settings, special attention should be given to the skeletal side effects of medications in such populations, and individualized management strategies should be implemented based on patient-specific factors.

From a clinical management perspective, individualized medication strategies are essential for high-risk groups. For older adults and individuals with higher body weight, enhanced monitoring of drug usage is recommended. In particular, patients on long-term pharmacotherapy should undergo regular bone mineral density assessments and prostate health evaluations, including serum prostate-specific antigen (PSA) testing and imaging studies, to ensure comprehensive monitoring (17). For patients with chronic gastrointestinal conditions requiring prolonged medication, safer alternative therapies should be prioritized, and adverse events should be closely monitored. Future drug development efforts should place greater emphasis on the safety profiles of medications in vulnerable populations to mitigate the incidence of drug-induced osteoporosis.

### 4.2 Sex differences in drug-induced osteoporosis

This study identified significant sex-based differences in drug-induced osteoporosis (DIO). Overall, the number of adverse event reports was notably higher in females, accounting for 64.6% of all cases, which aligns with the known epidemiological characteristics of osteoporosis (18). These sex differences may be attributed to variations in physiological structure, hormonal fluctuations, and differences in drug metabolism pathways. In particular, postmenopausal women are considered a high-risk population for DIO due to a rapid decline in estrogen levels and accelerated bone mineral loss (19). Additionally, women are often prescribed a wider range of medications—especially hormonal and antidepressant agents—which may further increase the incidence of bone metabolism disorders (20).

Among males, frequently reported potential signal drugs included tenofovir disoproxil (7,381 cases), esomeprazole (688), upadacitinib (368), adefovir (259), and tafamidis (200). Most of these drugs are known or suspected to interfere with bone metabolism, suggesting that males may also be at considerable risk of osteoporosis under specific pharmacological conditions. For instance, leuprorelin significantly reduces bone mineral density by suppressing androgen levels, while drugs such as tafamidis may contribute to systemic health deterioration that indirectly exacerbates bone loss (21–23).

In females, the most frequently reported drugs were tenofovir disoproxil (3,188 cases), esomeprazole (2,299), upadacitinib (1,507), tocilizumab (1,380), and ibrutinib (880). Some of these agents, including tocilizumab and upadacitinib, can disrupt bone remodeling processes by modulating immune mediators, cytokine signaling, or hormonal pathways, thereby increasing the risk of osteoporosis. Moreover, aromatase inhibitors such as anastrozole and letrozole can accelerate bone resorption by suppressing estrogen synthesis, posing a particular risk for postmenopausal women (24, 25).

Sex hormones play a critical role in bone metabolism regulation, and disruptions in estrogen or androgen levels can lead to significant bone loss. Drugs that interfere with endocrine pathways may induce different skeletal adverse effects in males and females. For example, glucocorticoids, aromatase inhibitors, and anti-androgen therapies may exert varying degrees of impact and mechanisms of action depending on the patient's sex. Therefore, when assessing the risk of drug-induced osteoporosis, it is essential to account for sex-specific characteristics and hormone sensitivity to inform personalized clinical prevention and intervention strategies (26–28).

### 4.3 Analysis of potential risk drugs

We further compared the signal detection results with official drug labeling and found that several drugs exhibited positive signals for osteoporosis in spontaneous reports, although no osteoporosis-related risks were explicitly listed in their product information. In total, 20 potential risk drugs were identified in the male population, with the most frequently reported being tenofovir disoproxil (7,381 cases), esomeprazole (688), upadacitinib (368), adefovir (259), and tafamidis (200). Among females, 28 potential risk drugs were identified, with top reported agents including tenofovir disoproxil (3,188 cases), esomeprazole (2,299), upadacitinib (1,507), tocilizumab (1,380), and ibrutinib (880).

Specifically, five drugs in males—upadacitinib^*^, tafamidis^*^, radium Ra 223 dichloride^*^, rivastigmine^*^, and lamivudine^*^–had more than 100 reported events. In females, eight drugs—esomeprazole^*^, upadacitinib^*^, tocilizumab^*^, octreotide^*^, leflunomide^*^, asfotase alfa^*^, aclidinium^*^, and imiglucerase^*^–exceeded 100 reports. These drugs represent novel osteoporosis signals not explicitly stated in the corresponding drug labels, suggesting the possibility of previously underrecognized bone metabolism-related adverse effects in real-world settings.

These agents may affect bone homeostasis via diverse mechanisms. For instance, upadacitinib and tocilizumab, as a Janus kinase (JAK) inhibitor and interleukin-6 (IL-6) pathway antagonist, respectively, may impair the balance between osteoclast and osteoblast activity during immune modulation, contributing to bone loss (29). Esomeprazole, a proton pump inhibitor, may reduce calcium absorption through long-term acid suppression, thereby lowering bone mineral density and increasing fracture risk (30). Although tafamidis is used for transthyretin amyloidosis, it may indirectly promote bone loss due to its association with impaired mobility and nutritional status. Enzyme replacement therapies such as asfotase alfa and imiglucerase may affect bone mineralization by altering phosphate or lipid metabolism.

These findings suggest that certain drugs without labeled osteoporosis warnings may still pose a significant risk of bone metabolic abnormalities during long-term use. In clinical practice, particularly among older adults, postmenopausal women, and patients with preexisting bone disorders, it is crucial to strengthen adverse event monitoring and routinely assess bone mineral density when prescribing these medications.

### 4.4 Time-to-onset analysis and risk management

This study analyzed the time-to-onset (TTO) characteristics of adverse events (AEs) associated with drug-induced osteoporosis, revealing distinct temporal patterns across different drugs and between sexes. In males, the drugs associated with the highest TTO risk included tenofovir disoproxil, esomeprazole, upadacitinib, adefovir, and tafamidis. Among these, tenofovir disoproxil exhibited the highest median TTO (1,555 days), indicating a strong propensity for delayed-onset AEs and substantial interindividual variability. In females, tenofovir disoproxil also showed the longest median TTO (1,698 days), similarly suggesting a delayed manifestation of osteoporosis-related events.

We further conducted Weibull distribution modeling to characterize the failure patterns of each drug. A shape parameter β < 1 with 95% CI entirely below 1 indicates an early failure pattern, where AEs tend to occur predominantly at the beginning of treatment. This was observed for upadacitinib in both males (β = 0.99, CI: 0.86–1.11) and females (β = 0.87, CI: 0.82–0.93), as well as tocilizumab (β = 0.76) and ibrutinib (β = 0.85) in females, suggesting a high-risk window during the early treatment phase.

Drugs with β approximately equal to 1 and 95% CI including 1 represent a random failure pattern, meaning the occurrence of AEs is not strongly time-dependent. This was found in adefovir (β = 1.22, CI: 0.86–1.58) and tafamidis (β = 1.10, CI: 0.73–1.47) in males.

In contrast, drugs with β > 1 and 95% CI entirely above 1 exhibit a wear-out failure pattern, indicating that the risk of AEs increases with prolonged exposure. This pattern was seen in tenofovir disoproxil (β = 1.18 in males; β = 1.21 in females) and esomeprazole (β = 1.32 in males; β = 1.15 in females), implying cumulative risk with long-term use.

These results have important implications for clinical risk management. For drugs exhibiting early failure patterns, such as upadacitinib, tocilizumab, and ibrutinib, enhanced monitoring is recommended during the initial treatment phase to promptly detect AEs. For drugs with wear-out failure characteristics, such as tenofovir disoproxil and esomeprazole, clinicians should implement long-term monitoring strategies, including periodic bone mineral density assessments, particularly in older adult patients or those with pre-existing bone conditions. Such individualized pharmacovigilance approaches may help mitigate the cumulative risk of osteoporosis associated with chronic drug use (31, 32).

### 4.5 Limitations of the study

Although this study leveraged a large volume of pharmacovigilance data from the FAERS database, several important limitations should be acknowledged.

First, FAERS is a spontaneous reporting system that inherently suffers from underreporting, selective reporting, and variable data quality. These characteristics may introduce substantial reporting bias and potentially lead to either overestimation or underestimation of drug-associated risks. Additionally, the database lacks detailed clinical and demographic information, including drug exposure details (such as whether the patient truly received the medication, at what dose, for how long, and under what conditions). This limits our ability to confirm the causality of the reported adverse events, as the suspected drug listed in a report may not necessarily be the true causative agent.

Second, the FAERS database does not provide data on disease severity, treatment adherence, or pharmacodynamic mechanisms. This lack of mechanistic data, along with the absence of critical clinical variables like bone mineral density assessments, comorbidities, and lifestyle factors, further limits our ability to make definitive conclusions regarding the biological mechanisms underlying drug-induced osteoporosis.

Third, a significant limitation of our study is the high proportion of missing data, particularly for key variables such as patient age and body weight, with 74% of the weight data missing. Due to the extent of missing data, we refrained from conducting further statistical analyses involving these variables. We did not apply any special handling techniques, such as imputation, as doing so could have introduced bias. The absence of these critical variables meant that we were unable to adjust for important confounders, such as age and body weight, which are known to significantly influence osteoporosis risk. Although we conducted stratified analyses by sex, the potential for residual confounding remains, and the observed sex-based differences in drug-osteoporosis associations should be interpreted with caution.

Finally, the observed sex-specific differences in osteoporosis risk may reflect variations in clinical decision-making, healthcare utilization, or reporting practices, rather than intrinsic biological susceptibility. The higher frequency of osteoporosis-related reports in females, for example, could be due to differences in prescribing patterns, as certain medications may be more commonly prescribed to women, leading to higher reporting rates in this population.

In light of these limitations, our findings should be considered exploratory and hypothesis-generating. Future research employing well-designed prospective cohorts, electronic health record-based data, randomized controlled trials, or mechanistic laboratory studies is warranted to validate these pharmacovigilance signals and to clarify the causal and sex-specific pathways involved in drug-induced osteoporosis.

## 5 Conclusion

This study investigated sex-specific differences in drug-induced osteoporosis (DIOP) using real-world pharmacovigilance data from the FAERS database. By applying a rigorous signal detection framework incorporating ROR, MGPS, and BCPNN, we identified high-confidence and previously undocumented risk signals, many of which were sex-specific.

Time-to-onset and Weibull modeling further distinguished risk patterns by drug and sex. Tenofovir disoproxil consistently exhibited a wear-out failure profile, suggesting cumulative toxicity and highlighting the need for long-term monitoring. In contrast, drugs such as upadacitinib, tocilizumab, and ibrutinib showed early failure tendencies, requiring vigilance during the initial treatment phase.

While our findings suggest that biological sex may influence drug safety profiles, this study did not assess pharmacodynamic mechanisms directly. The observed differences in signal strength and onset patterns may reflect underlying sex-related physiological factors, but further experimental studies are needed to confirm causality.

Given the limitations of spontaneous reporting systems—such as the inability to verify drug exposure, dose, and causality—these results should be interpreted cautiously. Nonetheless, the study provides actionable insights for personalized pharmacovigilance and underscores the need for sex-aware risk mitigation strategies in osteoporosis prevention and management.

## Data Availability

The original contributions presented in the study are included in the article/Supplementary material, further inquiries can be directed to the corresponding author.
