# Supplementary material for: Sex differences in drug-induced osteoporosis: a pharmacovigilance study based on the FAERS database
Source: Front Public Health. 2025 Jul 24;13:1630412. doi: 10.3389/fpubh.2025.1630412 (PMC12328446; doi:10.3389/fpubh.2025.1630412)
Supplement: Supplementary file 1 [file Table_1.docx]

**Supplementary material 1** Two-by-two contingency table for disproportionality analyses.

|  | Target AEs | Other AEs | Total |
| --- | --- | --- | --- |
|  | a | b | a+b |
| Other drugs | c | d | c+d |
| Total | a+c | b+d | a+b+c+d |

Abbreviation: AEs, adverse events; a, number of reports containing both the target drug and target adverse drug reaction; b, number of reports containing other adverse drug reaction of the target drug; c, number of reports containing the target adverse drug reaction of other drugs; d, number of reports containing other drugs and other adverse drug reactions.
