# Supplementary material for: Sex differences in drug-induced osteoporosis: a pharmacovigilance study based on the FAERS database
Source: Front Public Health. 2025 Jul 24;13:1630412. doi: 10.3389/fpubh.2025.1630412 (PMC12328446; doi:10.3389/fpubh.2025.1630412)
Supplement: Supplementary file 2 [file Table_2.docx]

**Supplementary material 2** 69 drugs related to osteoporosis

| DRUG | Drug classification | a | ROR | RORL | RORU | pvalue | p_adjust |
| --- | --- | --- | --- | --- | --- | --- | --- |
| TENOFOVIR DISOPROXIL* | Nucleoside Reverse Transcriptase Inhibitors (NRTIs) | 10924 | 86.07 | 83.75 | 88.44 | 0 | 0 |
| LENALIDOMIDE* | Immune Modulators | 5834 | 1.33 | 1.29 | 1.36 | 9.9354E-101 | 1.60556E-97 |
| ESOMEPRAZOLE | Proton Pump Inhibitors (PPIs) | 3669 | 4.38 | 4.24 | 4.53 | 0 | 0 |
| INTERFERON BETA-1A* | Immune Modulators | 3215 | 1.64 | 1.59 | 1.7 | 4.9591E-172 | 8.0139E-169 |
| TOFACITINIB | Tyrosine Kinase Inhibitors (TKIs) | 2750 | 1.71 | 1.65 | 1.78 | 1.2134E-173 | 1.9609E-170 |
| METHOTREXATE | Antimetabolites | 2370 | 1.45 | 1.39 | 1.51 | 3.52851E-71 | 5.70208E-68 |
| ABATACEPT* | Immune Modulators | 2081 | 2.01 | 1.92 | 2.1 | 5.5568E-224 | 8.9798E-221 |
| UPADACITINIB* | Tyrosine Kinase Inhibitors (TKIs) | 1923 | 3.05 | 2.92 | 3.2 | 0 | 0 |
| RITUXIMAB* | Monoclonal Antibodies (mAbs) | 1778 | 1.36 | 1.29 | 1.42 | 5.39765E-37 | 8.72261E-34 |
| PREGABALIN* | Neuroregulators | 1755 | 1.2 | 1.14 | 1.26 | 6.97507E-14 | 1.12717E-10 |
| TOCILIZUMAB* | Monoclonal Antibodies (mAbs) | 1612 | 2.27 | 2.16 | 2.38 | 1.0551E-241 | 1.705E-238 |
| IBRUTINIB* | Kinase Inhibitors | 1534 | 1.85 | 1.75 | 1.94 | 2.3446E-127 | 3.7889E-124 |
| LEUPRORELIN | Gonadotropin-Releasing Hormone (GnRH) Analogues | 1490 | 1.62 | 1.54 | 1.71 | 2.94119E-77 | 4.75297E-74 |
| PALBOCICLIB* | CDK4/6 Inhibitors | 1215 | 1.25 | 1.18 | 1.32 | 1.02435E-14 | 1.65535E-11 |
| OXYBATE SODIUM | GABA B Receptor Agonists | 1143 | 1.44 | 1.36 | 1.52 | 4.94955E-34 | 7.99847E-31 |
| RISANKIZUMAB* | Interleukin-23 (IL-23) Antagonists | 1086 | 1.68 | 1.58 | 1.78 | 4.69287E-65 | 7.58368E-62 |
| ANASTROZOLE | Aromatase Inhibitors (AIs) | 866 | 4.93 | 4.61 | 5.29 | 0 | 0 |
| ROFECOXIB* | Cyclooxygenase-2 (COX-2) Inhibitors | 771 | 1.71 | 1.59 | 1.83 | 8.7747E-50 | 1.41799E-46 |
| GOLIMUMAB* | Tumor Necrosis Factor-α (TNF-α) Antagonists | 724 | 1.21 | 1.13 | 1.31 | 2.50627E-07 | 0.000405013 |
| MACITENTAN* | Endothelin Receptor Antagonists (ERAs) | 719 | 1.52 | 1.41 | 1.63 | 1.48866E-28 | 2.40568E-25 |
| BOSENTAN* | Endothelin Receptor Antagonists (ERAs) | 718 | 1.38 | 1.28 | 1.48 | 1.68271E-17 | 2.71926E-14 |
| MEDROXYPROGESTERONE | Progestogens | 715 | 2.43 | 2.26 | 2.62 | 9.4001E-129 | 1.5191E-125 |
| LETROZOLE | Aromatase Inhibitors (AIs) | 703 | 2.82 | 2.62 | 3.04 | 1.7413E-174 | 2.814E-171 |
| OCTREOTIDE* | Somatostatin Analogues | 696 | 2.25 | 2.08 | 2.42 | 3.1364E-103 | 5.06837E-100 |
| PIMAVANSERIN* | 5-HT2A Receptor Antagonists | 668 | 1.39 | 1.29 | 1.5 | 2.50509E-17 | 4.04823E-14 |
| CELECOXIB | Cyclooxygenase-2 (COX-2) Inhibitors | 652 | 1.27 | 1.17 | 1.37 | 2.69232E-09 | 4.35079E-06 |
| OMEPRAZOLE | Proton Pump Inhibitors (PPIs) | 628 | 1.28 | 1.18 | 1.38 | 1.28374E-09 | 2.07453E-06 |
| RIVASTIGMINE* | Cholinesterase Inhibitors | 560 | 3.11 | 2.86 | 3.39 | 3.5827E-169 | 5.7897E-166 |
| LEFLUNOMIDE* | Immunosuppressants | 489 | 2.52 | 2.31 | 2.76 | 2.82287E-96 | 4.56176E-93 |
| VALSARTAN* | Angiotensin II Receptor Antagonists (ARBs) | 461 | 1.48 | 1.35 | 1.62 | 8.60461E-17 | 1.3905E-13 |
| THALIDOMIDE* | Immune Modulators | 446 | 1.41 | 1.29 | 1.55 | 3.85596E-13 | 6.23123E-10 |
| ADEFOVIR* | Nucleoside Analogues | 392 | 21.16 | 18.93 | 23.66 | 0 | 0 |
| ABALOPARATIDE* | Parathyroid Hormone Analogues | 364 | 1.65 | 1.48 | 1.83 | 2.59881E-21 | 4.19967E-18 |
| RIBOCICLIB* | CDK4/6 Inhibitors | 331 | 1.54 | 1.38 | 1.72 | 5.57063E-15 | 9.00214E-12 |
| PHENYTOIN | Antiepileptic Drugs (AEDs) | 323 | 1.65 | 1.48 | 1.84 | 2.73841E-19 | 4.42527E-16 |
| ASFOTASE ALFA* | Enzyme Replacement Therapy (ERT) | 314 | 3.17 | 2.83 | 3.55 | 6.72664E-99 | 1.08703E-95 |
| PANCREATIN* | Pancreatic Enzyme Replacement Therapy (PERT) | 293 | 2 | 1.78 | 2.24 | 1.44104E-32 | 2.32872E-29 |
| TAFAMIDIS* | Transthyretin Stabilizers | 260 | 1.93 | 1.71 | 2.18 | 2.87763E-26 | 4.65025E-23 |
| FULVESTRANT* | Selective Estrogen Receptor Modulators (SERMs) | 241 | 1.86 | 1.63 | 2.11 | 7.80622E-22 | 1.26149E-18 |
| EXEMESTANE | Aromatase Inhibitors (AIs) | 238 | 2.55 | 2.24 | 2.9 | 9.6756E-49 | 1.56358E-45 |
| IMIGLUCERASE* | Glycogen Storage Disease Enzyme Replacement Therapy | 215 | 4.24 | 3.69 | 4.86 | 7.253E-111 | 1.1721E-107 |
| ACLIDINIUM* | Anticholinergic Drugs | 214 | 4.04 | 3.52 | 4.64 | 1.4407E-102 | 2.32823E-99 |
| DIGOXIN* | Cardiac Glycosides | 208 | 1.61 | 1.4 | 1.85 | 9.87987E-12 | 1.59659E-08 |
| LAMIVUDINE* | Nucleoside Reverse Transcriptase Inhibitors (NRTIs) | 206 | 1.56 | 1.36 | 1.79 | 2.2553E-10 | 3.64456E-07 |
| ROTIGOTINE* | Dopamine Receptor Agonists | 199 | 1.94 | 1.69 | 2.24 | 6.79697E-21 | 1.09839E-17 |
| RANOLAZINE* | Antianginal Drugs | 186 | 1.47 | 1.27 | 1.7 | 1.73913E-07 | 0.000281044 |
| RADIUM RA 223 DICHLORIDE* | Bone-Targeted Radioisotopes | 177 | 3.17 | 2.73 | 3.69 | 1.98029E-56 | 3.20015E-53 |
| CORTICOTROPIN | Adrenocorticotropic Hormone (ACTH) Stimulants | 148 | 1.51 | 1.29 | 1.78 | 6.23763E-07 | 0.001008001 |
| DEFLAZACORT | Glucocorticoids | 144 | 3.47 | 2.94 | 4.1 | 3.74767E-54 | 6.05624E-51 |
| TOLTERODINE* | Anticholinergic Drugs | 126 | 1.86 | 1.56 | 2.22 | 4.50115E-12 | 7.27386E-09 |
| RASAGILINE* | Monoamine Oxidase B (MAO-B) Inhibitors | 115 | 3.43 | 2.84 | 4.13 | 1.37715E-42 | 2.22548E-39 |
| TRIPTORELIN* | Gonadotropin-Releasing Hormone (GnRH) Analogues | 115 | 2.24 | 1.86 | 2.7 | 3.34247E-18 | 5.40143E-15 |
| VELAGLUCERASE ALFA* | Enzyme Replacement Therapy (ERT) | 108 | 5.1 | 4.2 | 6.2 | 8.21788E-74 | 1.32801E-70 |
| ELOSULFASE ALFA | Enzyme Replacement Therapy (ERT) | 105 | 1.77 | 1.46 | 2.15 | 5.77675E-09 | 9.33523E-06 |
| GOSERELIN | Gonadotropin-Releasing Hormone (GnRH) Agonists | 103 | 1.57 | 1.29 | 1.91 | 5.98819E-06 | 0.009676914 |
| EFGARTIGIMOD ALFA* | FcRn Inhibitors | 96 | 2.46 | 2.01 | 3.02 | 5.42315E-19 | 8.76381E-16 |
| TENOFOVIR | Nucleoside Analog Reverse Transcriptase Inhibitors (NARTIs) | 91 | 4.06 | 3.29 | 5.02 | 1.04651E-44 | 1.69116E-41 |
| INDACATEROL* | Long-Acting Beta-2 (LABA) Agonists | 71 | 2.11 | 1.67 | 2.67 | 3.59592E-10 | 5.81101E-07 |
| GALANTAMINE* | Cholinesterase Inhibitors | 68 | 1.77 | 1.4 | 2.26 | 3.14896E-06 | 0.005088723 |
| CICLESONIDE | Glucocorticoids | 65 | 3.29 | 2.57 | 4.22 | 6.52946E-23 | 1.05516E-19 |
| ETELCALCETIDE* | Calcium-Sensing Receptor (CaSR) Agonists | 65 | 2.06 | 1.61 | 2.64 | 7.20306E-09 | 1.16401E-05 |
| AMIFAMPRIDINE* | Potassium Channel Blockers | 62 | 2.82 | 2.19 | 3.63 | 1.41461E-16 | 2.28601E-13 |
| CARBIDOPA* | Dopa Decarboxylase Inhibitors | 48 | 2.79 | 2.09 | 3.71 | 8.0465E-13 | 1.30031E-09 |
| ENTACAPONE* | COMT Inhibitors | 47 | 2.18 | 1.63 | 2.91 | 1.29004E-07 | 0.000208471 |
| DAPRODUSTAT* | Hypoxia-Inducible Factor Prolyl Hydroxylase (HIF-PH) Inhibitors | 42 | 3.81 | 2.8 | 5.19 | 3.23825E-19 | 5.23301E-16 |
| SATRALIZUMAB* | Interleukin-6 (IL-6) Receptor Antagonists | 34 | 2.97 | 2.11 | 4.18 | 1.61426E-10 | 2.60864E-07 |
| TALIGLUCERASE ALFA* | Enzyme Replacement Therapy (ERT) | 24 | 4.26 | 2.82 | 6.43 | 2.37514E-13 | 3.83822E-10 |
| FERRIC HYDROXIDE* | Iron Supplements | 15 | 7.46 | 4.39 | 12.67 | 7.33324E-09 | 1.18505E-05 |

ROR, reporting odds ratio; CI, confidence interval; FAERS, FDA Adverse Event Reporting System; P-adjust, p-value after Bonferroni correction; P-adjust<0.01, statistically significant; *New signals of osteoporosis adverse reactions are not mentioned in the instructions.
